# Effectiveness of a program for the development of socio-emotional competences in people admitted to a penitentiary center

**DOI:** 10.3389/fpubh.2022.1116802

**Published:** 2023-01-10

**Authors:** Lucía Granados, Raquel Suriá, Carles Perea, Claudio Payá, Laura Sánchez-Pujalte, David Aparisi

**Affiliations:** ^1^Faculty of Education, Valencian International University, Valencia, Spain; ^2^Department of Communication and Social Psychology, University of Alicante, Alicante, Spain; ^3^Faculty of Education, Carlemany University, San Julián de Loria, Andorra

**Keywords:** incarceration, reintegration, rehabilitation, emotional competences, emotional education

## Abstract

The purpose of this paper was to evaluate the effectiveness of a program for the development of social and emotional competences and self-esteem among a group of inmates at a penitentiary center, as well as to determine the possible correlation between the variables of the program (social skills, emotional competences, and self-esteem). The objective was to equip inmates with social competences in emotional regulation strategies that would be useful to them in the penitentiary center and, at the same time, facilitate their future social inclusion. In order to measure the pre- and post- treatment variables, the Social Skills Scale, the Perceived Emotional Intelligence Scale (TMMS-24), and the Rosenberg Self-Esteem Scale (RSES) were administered to a group of 51 inmates in a penitentiary center. The experimental group consisted of 29 inmates, with 21 forming the control group. The pretest-posttest ANOVAs showed that the program led to a significant (*p* < 0.01) increase in: (1) positive social behaviors; (2) emotional competences; (3) self-esteem. Positive correlations were also observed between the three variables. The results suggest the importance of implementing programs for the promotion of the socio-emotional development of people incarcerated in penitentiary centers.

## Introduction

Being admitted to a penitentiary center is a difficult and complicated process that involves living outside the society in which one has been a participant and starting a new life, which generates uncertainty and distrust. It is at this moment that a set of mechanisms for social reintegration (activities and programs) start to take action; the fundamental objective of which is to reintegrate the individual into society (1).

Incarceration is a process that inevitably affects all aspects of a person's life. In prison, inmates have to live with other people with very different characteristics, which creates an atmosphere of conflict that is difficult to avoid. The conditions of incarceration may lead to a series of psychological reactions, generated by permanent emotional stress (2–7), which may result in irreparable consequences for some vital aspects of a person's life (physical, psychological, social, emotional, professional...).

For example, prison demands that those admitted put in a great personal effort to be able to adapt well to an institution that is characterized by uncompromising isolation, an inflexible environment, constant surveillance, a lack of intimacy, and frustrating situations, which, among other things, condition interpersonal relationships based on distrust and aggressiveness. Similarly, the lack of contact with the family, the loss of daily habits and socio-labor and leisure routines, as well as the integration into a restrictive and deprived environment and social isolation can cause a significant deterioration in the emotional and social competences of inmates (8, 9).

These factors may lead to the emergence of psychological, emotional, and social imbalances for those affected (8–10). In this regard, different studies from around the world have shown that the prevalence rate of mental disorders in the prison population found in this study is 5.3 times higher than that of the general population, indicating that nine out of 10 inmates present some type of alteration in their mental health; rates range from 2 to 4% in disorders such as schizophrenia, 10 to 12% in depressive disorder, and 50 to 75% in personality disorders and psychological disorders (11).

Most authors have noted that inmates in penitentiary centers present a spectrum of psychological imbalances such as depression, anxiety, psychosis, personality disorders, substance abuse, and an increased risk of suicide as well as problems related to impulsivity, emotional self-control, difficulty in developing abstract thinking, difficulties in identifying and adequately solving interpersonal problems, low resistance to frustration, low self-esteem, among others (10, 12–15).

Given the need to reduce the risk of recidivism, mitigate all the possible negative health effects, and fully promote the re-education and subsequent labor and psychosocial reintegration of inmates, the scientific community has designed a variety of treatment programs for the prevention of criminal behavior (2, 15–20). There are many models in the literature that have tried to provide an answer to criminal behavior, with two particular ones standing out: the RxNxR (risk-need-responsivity) model by Andrews and Bonta (21) and the GLM (Good Lives Model) by Ward et al. (20). The first model, designed by Andrews and Bonta (21), attempts to explain the individual differences in criminal behavior by addressing the influences in the closest social, cultural, and family context, as well as the individual's personal variables (biological, psychological, cognitive, behavioral, educational, etc.). Therefore, to reduce delinquency, it is a person's context and attitudes and habits that must be modified.

The second model, designed by Ward et al. (20), proposes a prison re-education theory using the legislative, ethical, and criminological framework of human rights. These rights help to discern the most basic human needs of prisoners, identify appropriate lifestyles, and facilitate prison environments that are more respectful and humane to prisoners (17). Therefore, this model proposes moving from a therapeutic and rehabilitative view of human rights in general to the specific right to education and teaching. As such, as an alternative, this approach offers an idea that is based not only on the reduction of risk factors but also on enabling inmates with the resources they need to live a better lifestyle (20).

Based on the idea of re-education, the majority of treatment programs in prison have a cognitive-behavioral psychological approach, as this has been shown to have greater efficacy in various evaluative measures, which also includes the reduction of criminal recidivism (2, 17, 22–24). This approach is based on the general psychological principle that cognitive processes influence behavior. It is therefore considered that if a person changes their thoughts, attitudes, reasoning, and interpersonal problem-solving cognitive abilities (which also involves improving their emotional control and teaching them new skills and behaviors), it is more probable that they will experience prosocial behavior and a reduction in the frequency and severity of their criminal activities (15, 25).

In this sense, cognitive-behavioral interventions have been focused on the development of different skills that are found to be deficient, and therefore, more of a priority to develop in the inmate population (24, 26). In this way, we follow the work of Redondo and Mangot (27) and Casado and Ruano (2) who, through a review of in-depth studies on treatment programs for inmates in penitentiary centers, found that the most effective programs seem to be those that include techniques aimed at improving inmates' reasoning skills, empathy, the evaluation of their behaviors toward others and toward themselves, their ability to reflect before acting, their problem-solving skills, and also their generally underdeveloped social skills.

Among the most deficient competences, empirical evidence shows social skills as being necessary and lacking among inmates in correctional facilities (3–6, 15, 28). In the majority of situations, inmates do not possess the necessary social skills or competences to be able to manage the difficulties they face in life, respond assertively to problems and situations within the prison setting, and build on positive behaviors for their personal wellbeing, and, therefore, programs that focus on developing these abilities or skills are necessary. As a result, in these spaces, many difficulties with interpersonal relationships have been found, making social skills a key element for proper social functioning (29–31).

Inmates are people who, due to the circumstances they have experienced and are experiencing, present difficulties when reacting in a socially acceptable way, which generates harmful consequences, for both them and for their environment: verbal and physical aggression, conflicts, addictions, displacement, diseases (13, 32). This means that the development of an adequate level of emotional intelligence is a necessary requirement for this population (33–35).

Another necessary construct, and one that the published literature has highlighted as being a deficit in inmates in penitentiary centers, is the lack of awareness and poor management and regulation of emotions. Consequently, there are many authors that note that focusing on affective factors in the first stages of treatment improves the results achieved by such a treatment on inmates (22, 23, 32, 34).

Incarceration is a process that inevitably affects all aspects of a person's life. In prison, they have to live with other inmates with very different characteristics, which creates an atmosphere of conflict that is difficult to avoid.

Finally, an aspect that is very much related to these skills, and one that is currently included in psychosocial programs for prison treatment, is self-esteem (19, 36).

Using a broad psychological perspective, Rosenberg (37) considers self-esteem to be a fundamental component of self-awareness and defines it as being an overall positive or negative attitude that an individual has toward themself, that is, a set of feelings and thoughts about their own value and importance. Regarding incarceration, different studies have shown that prison significantly affects the self-esteem of inmates and leads them to generate negative beliefs about their self-image, creating low levels of self-esteem and self-perception (38, 39).

Considering the importance of addressing these deficits in the socio-emotional competences of inmates and the need for their development for adequate psychosocial and socio-emotional reintegration, it is necessary to have programs that include these three constructs in the behavioral resources of inmates. However, it can be noted in the literature on the treatment programs for criminals that the majority of studies have been focused in a specific way, that is, on the promotion of social skills (31), affective competences (13), or empathy (40), with programs that integrate the development of interventions for the promotion of these skills together therefore not being common.

Using the results found in the previous research, this present study aims to examine the effectiveness of a program based on techniques, competences, and socio-emotional skills designed to achieve a high degree of personal autonomy for the socio-emotional inclusion of inmates. Specifically, this study has two objectives. The first objective is to evaluate the effectiveness of the program in increasing the social skills, emotional competences, and self-esteem of a group of inmates in a penitentiary center. The second objective of the study is to determine the possible correlation between the variables of the program (social skills, emotional competences, and self-esteem).

## Methods

### Participants

The sample used in this study was comprised of a total of 51 male participants, with an average age of 35.22 (SD = 12.21). From this group, 29 participants made up the experimental group and 22 on the waiting list formed the control group. With regard to the sociodemographic profile of the sample, the majority of the participants had a literacy-level education (59.4%), were single (84.3%), and did not have children (76.5%). The distribution between the groups according to age, education, marital status, and offspring was homogeneous (*p* > 0.05) (Table 1). In terms of participation, it is worth noting that the sample consisted of a group of inmates that could be considered to be small in size. This was due to the difficulty in recruiting a larger number of participants for the study. This difficulty was a result of restricted access to some services of the penitentiary institution and to the increase in security and performance measures. All participants were inmates in compliance and prevention units. The participants volunteered to be involved after having been informed of the study and after being guaranteed anonymity.

**Table 1:** Sociodemographic characteristics of the groups.

		**Control**	**Experimental**	**Total**	***t/χ^2^***	**p**
Age		38.86 (12.18)	32.45 (11.68)	35.22 (12.21)	1.91	0.06
Studies	No studies	(14) (27.5%)	(14) (27.5%)	28 (59.4%)		
	Primary	(6) (11.8%)	(4) (7.8%)	10 (19.6%)		
	Professional training	(3) (5.9%)	(0) (0%)	3 (5.9%)	5.69	0.22
	Spanish Baccalaureate	(3) (5.9%)	(4) (7.8%)	7 (13.7%)		
	University	(3) (5.9%)	(0) (0%)	3 (5.9%)		
Civil Status	Single	(18) (35.3%)	(25) (49%)	43 (84.3%)		
	Married/with a partner	(2) (3.9%)	3 (5.9)	5 (9.8%)	0.73	0.69
	Divorced	(2) (3.9%)	(1) (2%)	3 (5.9%)		
Children	Has children	(8) (15.7%)	(4) (7.8%)	12 (23.5%)	3.54	0.06
	No children	(14) (27.5%)	(25) (49%)	39 (76.5%)		

### 2.2. Instruments

#### 2.2.1. The Trait Meta-Mood Scale-24

This instrument uses the Spanish adaptation (41) of the TMMS-48 created by Salovey et al. (42). The Spanish adaptation consists of 24 items that are responded to using a 5-point Likert scale (1 = Not at all agree; 5 = Strongly agree). The items are distributed among three scales: Emotional Attention, Emotional Clarity, and Emotional Repair. The scale is composed of three dimensions with eight items in each: Attention to one's own feelings, Emotional clarity, and Emotional repair. Emotional attention is defined as the ability to identify and express feelings in an appropriate way, Emotional clarity is the understanding of emotional states, and Emotional repair is the ability to regulate emotional states correctly. This test was chosen due to its easy administration, its satisfactory psychometric characteristics, and because it had been validated for the adult population (41); the Cronbach's alpha was higher than 0.85 in all three scales.

#### 2.2.2. The social skills checklist

The items come from other psychological studies that provide information about the right behaviors that allow individuals to behave correctly in different contexts. It is comprised of a total of 50 items with a Likert-type response format (1 = not at all, 5 = always), and grouped into six areas: basic social skills (listening, thanking, asking questions, etc.,), advanced social skills (asking for help, participating, apologizing, etc.,), skills related to emotions (expressing and understanding feelings, expressing affection, fears, etc.,), alternative skills to aggression (sharing, helping, self-control, etc.,), skills for dealing with stress (formulating and responding to complaints, defending others, etc.,), and skills for planning (taking initiatives, making decisions, setting goals, etc.,) (1). The main objective of this is to determine the deficiencies and competences that a person may have in their social skills and evaluate in what type of situations they are competent or deficient in the use of a social skill. The reliability and factorial structure of the instrument reveal the validity of the instrument in the adult population. Each one of the component scales obtained a highly significant and positive correlation at a level of *p* < 0.001 with the Total Social Skills Scale, as well as a very good internal consistency (α = 0.92).

#### 2.2.3. The Rosenberg Self-Esteem Scale

This is one of the most widely used scales for the overall measurement of self-esteem. The instrument consists of 10 items whose contents are focused on feelings of respect and acceptance of oneself. Half of the items are phrased positively and the other half negatively. Although it was initially designed as a Guttman scale, it has subsequently become common to use it as a Likert-type scale, where items are answered on a four-point scale (1 = strongly agree, 2 = agree, 3 = disagree, 4 = strongly disagree). In terms of correction, the scores of the negatively phrased items (3, 5, 8–10) are inverted and then all the items are added together. The score range of the questionnaire spans from 10 to 40, with a higher score indicating a higher level of self-esteem. The cut-off point in the adult population is 29 and the internal consistency alpha coefficient is 0.92.

#### 2.2.4. Intervention program

The intervention program aims to develop socio-emotional competences and skills with the objective of achieving a high degree of personal autonomy for the socio-emotional inclusion of the inmates. The modality used consists of theoretical-practical workshops, with a participative and critical attitude toward them being encouraged. The program consists of seven modules with a duration of 12 h for each one and two weekly sessions of 1 h and 30 min over 10 months, which are given by a psychologist in groups of 14 or 15 people. The methodology is totally practical and is done through socio-educational support and activities that aim to achieve the established objectives and work on the specific contents of the module in order to train the individuals with strategies and socio-emotional skills that will allow them to acquire greater socio-labor and personal insertion. The activities are carried out through debates, group dynamics, role-playing, and videos. The contents worked on in each module are the following:

Module 1. Emotional self-awareness: Definition and components of socio-emotional competence. Self-awareness: Conceptualization and components, the relationship of self-awareness to self-regulation and empathy. Emotions: Definition and components, emotional vocabulary, positive affects, and analysis of emotions. Sadness affects: Definition and characteristics, emotional transition, emotional states related to restlessness, emotional states related to anger.
Module 2. Emotional regulation: Emotional regulation, the process of emotional self-regulation, levels of interpersonal communication, attributional or thinking style, Ellis' irrational ideas, conflict resolution using the A-B-C technique, social and conversational skills, Avoidance, adaptive behaviors in positive situations, situations in which to work on emotional regulation.
Module 3. Empathy: Empathy, communication, empathic listening, empathic response, empathic understanding, empathic concern, social-emotional competences and professional development, empathy in professional development.
Module 4. Motivation: Motivation and related terms, main theoretical models on motivation: Psychoanalytic, behaviorist, Maslow, Mc Clelland, and others, motives, objectives, goals and expectations in motivation, the achievement and power motive: Fundamental characteristics, affiliative motives, avoidance motives, the role of self-efficacy in motivation, the role of attributional systems and self-efficacy in motivation, the treatment of motivation in organizations.
Module 5. Assertiveness: Assertiveness: definition and characteristics, assertive rights, assertiveness and self-esteem, the passive, assertive and aggressive response, the behavioral, cognitive and emotional component, type of assertive response: Basic, empathic, intense and compulsive, Assertiveness techniques.
Module 6. Teamwork: Group cohesion and its improvement, group objectives, productivity, maturity and value of the workgroup, self-perception, group work, and work groups.
Module 7. Conflict resolution: Conflict: concept, origin and scope, appropriate conflict management, attitudes, and different ways of dealing with conflict. Techniques and strategies for dealing with conflict. Mediation in conflicts.

### Data analysis

We carried out a quasi-experimental study with an experimental group, control group, intersubject factor, and an intrasubject factor (before and after treatment) with six levels for the social skills variables (basic social skills, advanced social skills, skills related to emotions, alternative skills to aggression, skills for dealing with stress, and skills for planning), three levels for emotional intelligence (attention, clarity, and repair), and one for self-esteem. Firstly, the *t* and χ^2^ tests were used to explore the distribution of age, studies, civil status, and number of children according to the experimental and control groups. Correlations between all variables were subsequently analyzed before the intervention. Finally, multivariate (MANOVA) and univariate (ANOVA) analyses were carried out, and the effect size of the differences was calculated using Cohen's *d* coefficient.

#### Permission to reuse and copyright

Permission must be obtained for use of copyrighted material from other sources (including the web). Please note that it is compulsory to follow figure instructions.

## Results

The correlations between the variables for the application of the program were significant and positive with a level of *p* < 0.001 in 21 of the 45 comparisons and with a value of *p* < 0.05 in five comparisons, with the main associations being concentrated between the social skills and emotional intelligence variables (see Table 2).

**Table 2:** Correlations between study variables.

	**1**	**2**	**3**	**4**	**5**	**6**	**7**	**8**	**9**	**10**
2	0.610^**^	1								
3	0.414^**^	0.522^**^	1							
4	0.260	0.406^**^	0.382^**^	1						
5	0.372^**^	0.067	0.395^**^	−0.058	1					
6	0.305^*^	0.393^**^	0.404^**^	0.209	0.534^**^	1				
7	−0.063	0.148	−0.078	0.315^*^	0.126	0.253	1			
8	0.221	0.373^**^	0.648^**^	0.193	0.211	0.186	0.233	1		
9	0.540^**^	0.645^**^	0.540^**^	0.304^*^	0.249	0.563^**^	0.080	0.258	1	
10	0.276^*^	0.454^**^	0.360^**^	0.169	0.375^**^	0.645^**^	0.231	0.187	0.541^**^	1

1, basic social skills; 2, advanced social skills; 3, skills related to emotions; 4, alternative skills to aggression; 5, skills for dealing with stress; 6, skills for planning; 7, self-esteem 8, attention; 9, clarity; 10, repair.

** *p* < 0.001,

* *p* < 0.05.

### 3.1. Social skills

The results showed that there were no differences in the scores for the social skills variables between the control and experimental groups before the application of the program *F*_(6,49)_ = 2.01, *p* = 0.085, but there were differences post-treatment *F*_(6,49)_ = 27.88, *p* < *0.0*01, η^2^*p* = 0.792 with there being a large magnitude in terms of the differences in all cases and the values of *d* ranging between 0.82 and 3.32.

In terms of the intragroup differences, in the experimental group there were statistically significant differences between stress levels before and after the treatment in all social skills variables and the magnitude of the differences were large for Basic social skills (*F* = 169.87, *p* < 0.001, *d* = 2.16), Skills related to emotions (*F* = 62.52, *p* < 0.001, *d* = 1.64), Alternative skills to aggression (*F* = 52.90 *p* < 0.001, *d* = 1.18), Planning Skills (*F* = 47.78, *p* < 0.001, *d* = 1.20), and there was a medium magnitude for Advanced social skills (*F* = 14.84, *p* = 0.001, *d* = 0.69) and Skills for dealing with stress (*F* = 35.15, *p* < 0.001, *d* = 0.69).

**Figure 1:**
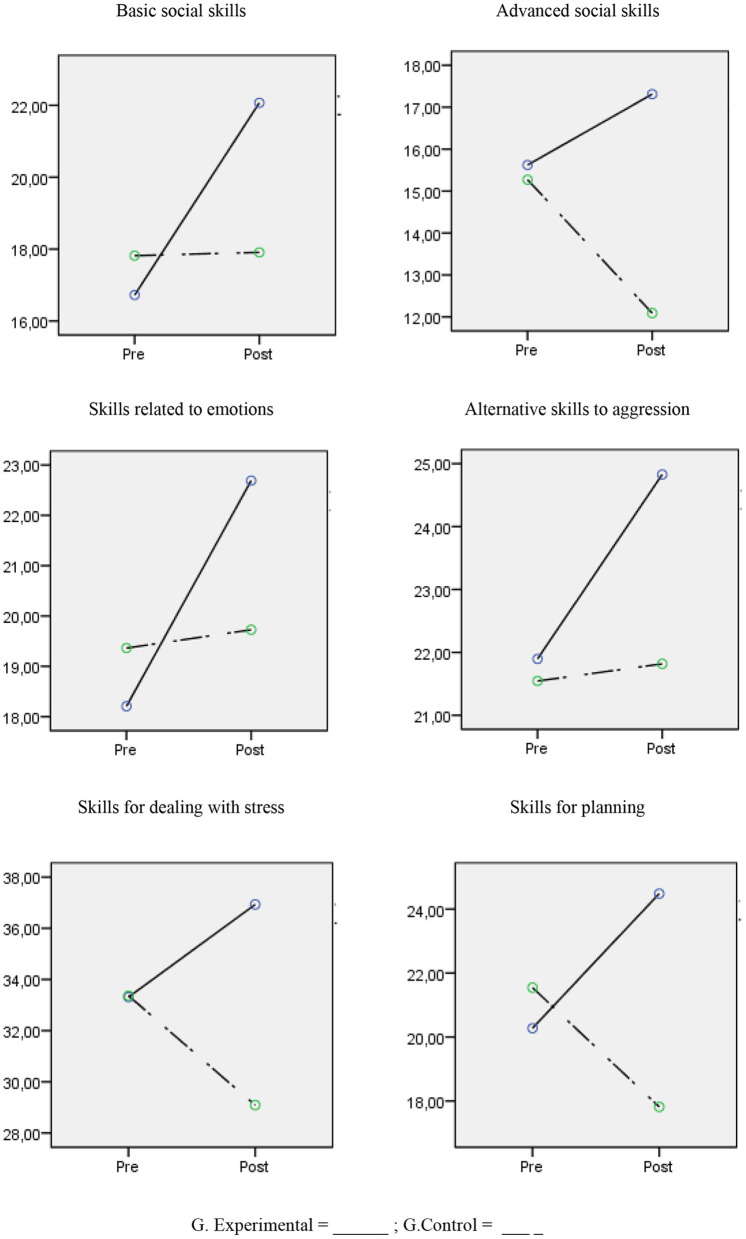
Change in social skills scores between experimental and control groups.

Regarding the control group, there were significant differences but these were shown in a reverse trend, with there being lesser scores in the post-treatment in Advanced social skills (*F* = 30.92, *p* < 0.001, *d* = 1.24) and Skills for dealing with stress (*F* = 27.35, *p* < 0.001, *d* = −0.95), and in the rest of variables no statistically significant differences were found (see Table 3; Figure 1).

**Table 3:** Means and standard deviations in social skills variables of pre- and post-treatment groups: a posteriori contrasts.

		**Experimental**		**Control**		**Significance** **statistic**		
**Dimensions**		***M***	**SD**	***M***	**SD**	***F***	***p***	***d***
1	Pre	16.72	2.20	17.81	3.11	2.16	0.14	–
	Post	22.06	2.72	17.90	2.28	33.90	<0.001	0.1.64
2	Pre	15.62	2.93	15.27	3.44	0.16	0.69	–
	Post	17.31	1.81	12.09	1.19	136.86	<0.001	3.32
3	Pre	18.20	3.20	19.36	4.52	1.15	0.29	–
	Post	22.69	2.16	19.72	4.09	11.15	0.00	0.95
4	Pre	21.89	2.79	21.54	4.72	0.11	0.74	–
	Post	24.82	2.15	21.81	4.99	8.50	0.00	0.82
5	Pre	33.31	4.87	33.36	5.03	0.001	0.97	–
	Post	36.93	4.06	29.09	3.93	47.84	<0.001	1.98
6	Pre	20.27	4.32	21.54	3.71	1.22	0.27	–
	Post	24.48	2.42	17.81	3.48	64.75	<0.001	2.48

1, basic social skills; 2, advanced social skills; 3, skills related to emotions; 4, alternative skills to aggression; 5, skills for dealing with stress; 6, skills for planning.

### 3.2. Emotional intelligence

**Figure 2:**
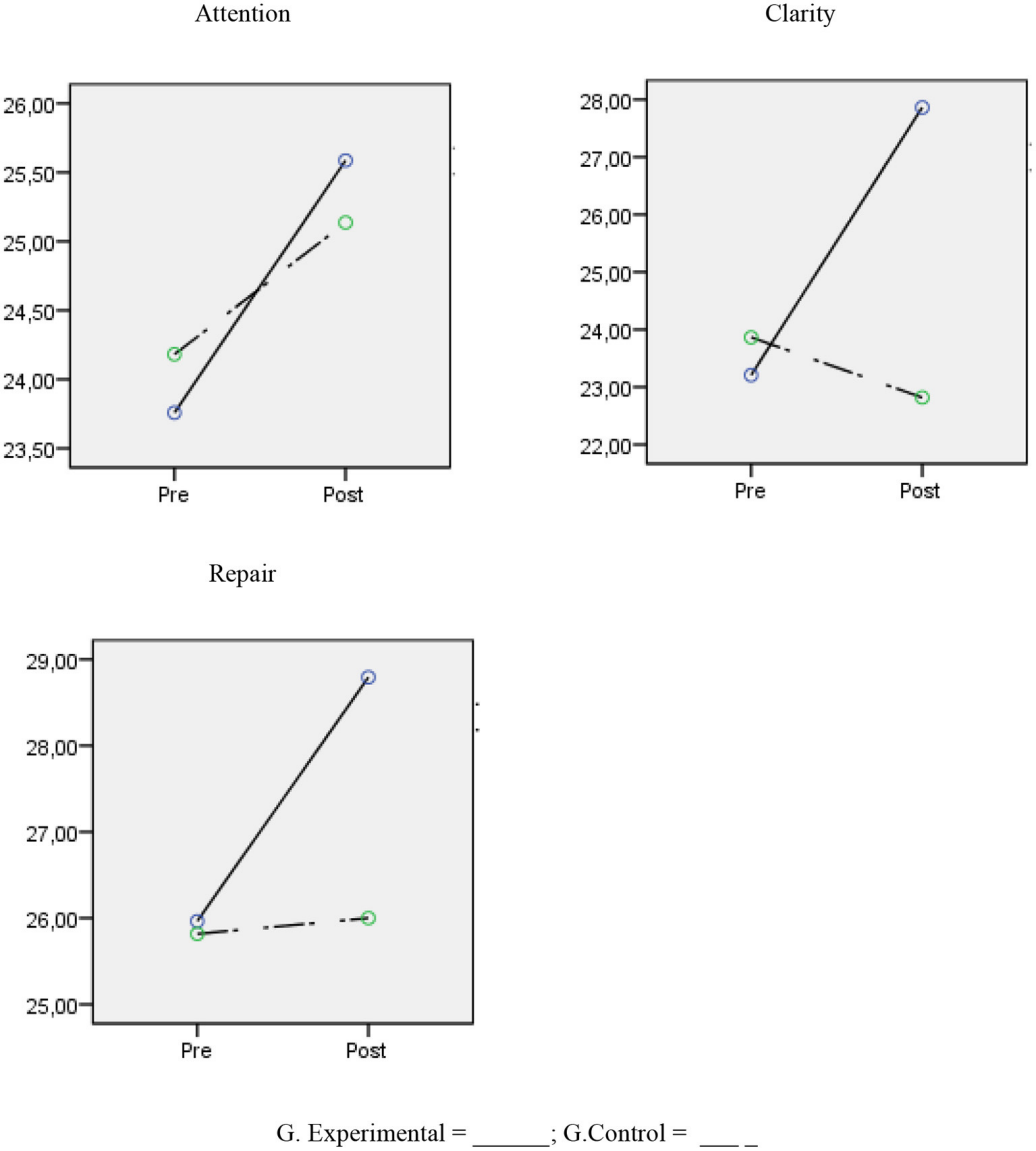
Change in emotional intelligence scores between experimental and control groups.

The results showed that there were no differences in the scores for the emotional intelligence variables between the control and experimental groups before the application of the program [*F*_(3,47)_ = 0.099, *p* = 0.960], but there were differences post-treatment [*F*_(3,47)_ = 3.07, *p* = 0.037, η^2^*p* = 0.164] with the experimental group scoring higher on the clarity variable with a large magnitude in terms of the differences (*d* = 0.86). In terms of the intragroup differences between the pre- and post- treatment scores, the scores for the experimental group were statistically significant and had a high magnitude for the clarity variable (*F* = 43.50, *p* < 0.001, *d* = 1.06), and had a small magnitude for the repair (*F* = 6.25, *p* = 0.019, *d* = 0.49) and attention (*F* = 5.99, *p* = 0.021, *d* = 0.28) variables. However, there were no differences for the same variables in the control group; attention (*F* = 1.87, *p* = 0.288), clarity (*F* = 2.35, *p* = 0.140), and repair (*F* = 0.06, *p* = 0.813) (see Table 4; Figure 2).

**Table 4:** Means and standard deviations in emotional intelligence variables of pre- and post-treatment groups: a posteriori contrasts.

		**Experimental**		**Control**		**Significación** **estad**í**stica**		
**Dimensions**		***M***	**SD**	***M***	**SD**	***F***	***p***	***d***
Attention	Pre	23.75	6.11	24.18	3.51	0.08	0.773	–
	Post	25.59	6.88	25.14	4.95	0.03	0.871	–
Clarity	Pre	23.20	5.04	23.86	7.17	0.15	0.703	–
	Post	27.86	3.64	22.81	7.91	9.25	0.004	0.86
Reparation	Pre	25.96	6.17	25.81	7.56	0.01	0.939	–
	Post	28.79	5.25	26.01	6.35	2.41	0.127	–

### Self-esteem

**Figure 3:**
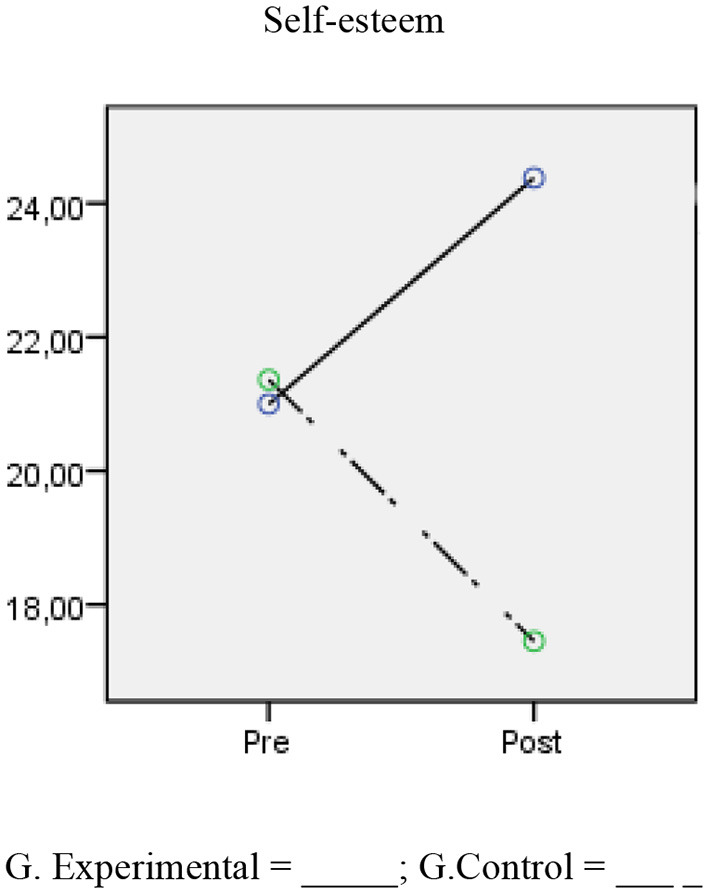
Change in self-esteem scores between experimental and control groups.

The results showed that there were no differences in the scores for the self-esteem variable between the control and experimental groups before the application of the program [*F*_(1,49)_ = 0.245, *p* = 0.623], but there were differences post-treatment [*F*_(1,49)_ = 157.79, *p* < 0.001, η^2^*p* = 0.76*, d* = 3.55]. In terms of the intrasubject differences between the pre- and post- treatment scores, the scores for the experimental group increased and were statistically significant and had a high magnitude for the self-esteem variable [*F*_(1,27)_ = 95.64, *p* < 0.001, *d* = 1.45], and regarding the control group, the differences were also significant and had a high magnitude but in the opposite direction [*F*_(1,27)_ = 0.72.63, *p* < 0.001, *d* = −1.73] (see Table 5; Figure 3).

**Table 5:** Means and standard deviations in self-esteem variable of pre- and post-treatment groups: a posteriori contrasts.

		**Experimental**		**Control**		**Statistic** **significance**		
**Dimensions**		***M***	**SD**	***M***	**SD**	***F***	***p***	***d***
Self-esteem	Pre	21.00	2.37	21.36	2.87	0.25	0.62	–
	Post	24.37	2.27	17.45	1.40	157.79	<0.001	3.55

## Discussion and conclusions

This study aims to identify the effectiveness of a program for the development of socio-emotional skills and competences aimed at people in the prison context. This type of program is important due to the relationship between incarceration and the poor emotional and social competences of inmates. With this serving as the context, the objective of the socio-educational program is to support and develop three fundamental pillars: social skills, emotional intelligence, and self-esteem.

By implementing and developing the program with the inmates under study, this paper establishes two objectives, with the first being the analysis of whether the program has been effective in increasing social skills, emotional competences, and self-esteem. As such, in terms of the effectiveness of the program in enhancing social competences, the results show that is effective in developing these skills in this population. Thus, higher scores are observed in the experimental group when compared with those of the control group after the program in all dimensions of social skills. These changes do not only vary between the control group and the experimental group post-treatment but also in terms of the differences found for the experimental group before and after the program, with there being greater differences after the intervention program.

These results show that the implementation of the program allows inmates to improve in different social skills (basic social skills, advanced social skills, skills related to emotions, alternative skills to aggression, skills for dealing with stress, skills for planning). Regarding the magnitude of the differences, an increased effect size can be mainly seen in the skills related to emotions, skills for dealing with stress, and skills for planning when comparing the mean scores of the experimental group after the program.

These changes are in line with the previous findings in the incarcerated population with regard to the effectiveness of cognitive-behavioral programs for inmates for the development of better interactions with their peers and with facility professionals, and, therefore, also for the development of the social skills they have in their behavioral repertoire (15, 29–31, 43, 44).

An interesting result that was found in the control group after the implementation of the program is the reduction of social skills. This result suggests that the passage of time in penitentiary centers, as a result of depriving individuals of their freedom, inhibits their social ties and behaviors and reduces their social interactions, which consequently has an impact on normalized interpersonal relationships. These results are supported by empirical evidence on the effects of prison on inmates, which indicates somatic consequences (sensory problems), changes in personal image, and, mainly, psychosocial consequences caused by having their freedom taken away, such as the loss of social skills (3–6, 15, 45, 46).

Regarding the results of the program for the development of emotional intelligence, the analyses show that the program contributed to the improving the three dimensions that make up emotional intelligence, that is, attention, clarity, and emotional repair, in the experimental group when comparing the mean scores before the implementation of the program with those after the program. Similarly, in the comparisons between the experimental group and the control group, statistical differences were observed in the clarity dimension between the two groups after treatment.

These results support previous findings from other studies that have noted that socio-emotional intervention programs can increase emotional intelligence. In other words, they enable a person with the necessary skills to attend to their own emotions and the emotions of others, as well as regulate their emotions and, thus, help to reduce certain behaviors such as impulsivity and emotional harshness, as well as increase frustration tolerance and self-control, which are constructs that are protective factors against criminal behaviors (13, 23, 25, 34, 47).

In line with these results, Filella et al. (13) evaluated the effectiveness that the implementation of an emotional education program had for the improvement of attention, awareness, and emotional repair in a group of inmates at a penitentiary center. The authors found that an increase in the management and regulation of emotional competences in the population was necessary for the reduction of aggressive and impulsive behaviors and to encourage self-control. Similarly, Howells and Day (23), after examining the effects of an emotional regulation program, noted that increasing an individual's awareness of affective factors during confinement improves the outcomes of the program in violent offenders.

Other studies, such as the work by Roger and Masters (48) and the later work by Greer (34), which were more focused on the reduction of certain disruptive behaviors, implemented programs that included emotional regulation and control in order to reduce the impulsivity of aggressors. The results showed a significant reduction in impulsivity after the training, which was shown through much more adaptive attitudes and better subsequent insertion.

Regarding the effects of the program in terms of the development of self-esteem, the results show an increase in this construct when comparing the post-treatment scores obtained by the experimental group with those obtained by the control group. Similarly, higher scores were observed in the experimental group after the treatment when compared to the scores obtained before the implementation of the program. Similar results were found in the study by Echeburúa and Fernández-Montalvo (39), in which they examined the effectiveness of a psychological treatment program for men incarcerated for serious crimes. According to the results, they found a significant increase in self-esteem and a significant modification of cognitive biases and cognitive restructuring. At the same time, the inmates under study experienced a reduction in psychopathological symptoms of impulsivity and anger. Impulsivity and depressive symptoms before treatment were predictors of a therapeutic outcome. Another study with similar findings is that of Larrota et al. (14), who examined the association between self-esteem and coping strategies in a group of inmates. In their results, they found that higher scores in adequate coping strategies related to problem-solving with self-esteem, while emotional avoidance, aggressive behaviors, and denial were associated with low self-esteem. Other works with comparable lines of research find similar positive results in terms of the development of self-esteem for the development of prosocial behavior and inhibition of aggressive behaviors (5, 18, 19, 36).

The second objective of the paper was to examine the degree of association that exists between the three program variables, that is, social skills, emotional intelligence, and self-esteem. The results show high relationships between most of the components of the three constructs. In particular, high correlations between the dimensions of emotional competences referring to attention, clarity, and emotional repair can be seen with the dimensions of basic social skills, advanced skills, skills related to emotions, and skills for planning. These findings confirm the results obtained in other studies that have noted that people with developed emotional competences, that is, attention, clarity, and emotional repair, are characterized by having adequate social skills in their behavioral repertoire (4, 5, 15, 44), and self-regulated, prosocial, and empathic behaviors (13, 23, 34, 47).

The results of this work point in the same direction as the meta-analysis of Papalia et al., as well as the results found by other authors (15, 44) in terms of the effectiveness of psychoeducation programs aimed at improving social and emotional skills, attitudes, and behaviors. The changes resulting from the program in terms of the social and emotional development of inmates can be explained by the type of activities that they include as they stimulate emotional awareness, social skills, empathy, confidence, and constructive problem-solving (49).

Therefore, generally speaking, the results show an improvement in the psychoemotional and social quality of life of the participants, by allowing them to develop attitudes and behaviors that can help prevent future harmful situations such as antisocial, disruptive, or aggressive behavior, or the neglect of their health in general (50). The implementation of this type of intervention program can improve the quality of life of people who are already in the prison system and could also be very useful as a preventive measure, thereby averting their entry into the prison system (49, 51).

However, the study has some limitations. The main limitation is the sample size. It would be beneficial for future studies to include more participants and also study the findings in the long term in order to obtain more reliable and statistically significant results. It would also be interesting to analyze these results according to the variables associated with the reasons for incarceration and the degree to which the inmate completes their sentence. Finally, another limitation is the use of self-reports due to the social desirability biases associated with them. Future studies replicating this research should use an observational methodology and assessment instruments based on the recording of positive and negative social behaviors.

Despite these limitations, the paper has practical implications for the penitentiary, psychoeducational, and reintegration spheres, as it provides a tool for the promotion of social and emotional competences during incarceration (positive social behaviors, emotional intelligence, and self-esteem) based on the tests. The results found after implementing the program suggest the importance of these types of programs as an instrument for prevention and intervention that promotes the social and emotional development of inmates in penitentiary centers.

Finally, the results obtained in this study point to the importance of designing and implementing psychoeducational intervention programs aimed at developing and improving socioemotional skills and self-esteem in the prison population in order to facilitate their social re-inclusion, once the relationship between the variables studied has been proven.

## Data availability statement

The raw data supporting the conclusions of this article will be made available by the authors, without undue reservation.

## Ethics statement

The studies involving human participants were reviewed and approved by Comisión de Ética para la Investigación y la Docencia - CEID Vice-Rectorate for Research and Knowledge Transfer Universidad Internacional de Valencia - VIU. The patients/participants provided their written informed consent to participate in this study.

## Author contributions

LG and CPe conceived conceptualization and designed the methodology. RS, LS-P, CPa, and LG performed the resources, calculation, and investigation of the data. RS, DA, and LG: writing—review and editing. DA and LG: supervision. All authors have read and agree to the published version of the manuscript.
